# Glasgow Royal Infirmary

**Published:** 1831

**Authors:** 


### XXXIII.

#### GLASGOW ROYAL INFIRMARY.

SEM-ENNIAL REPORT. By Dr. PERRY.*

THIS report opens with the following statements. At the present time they may excite more interest than the most recondite speculations or surprising cases.

"The surgical duties of the Glasgow Royal Infirmary are committed to the care of four surgeons, two senior and two junior, each of whom, till about the year 1826, took them wholly upon himself for a period of three months, the others only attending when called in cases of consultation, or to operations. About the period mentioned, (the number of surgical patients having increased,) it was thought advisable that two surgeons should attend daily, each having the same number of wards, attending the waiting room, and admitting patients, week about, their period of attendance being extended to six months. This was a decided improvement upon the former system, giving the surgical attendants more time, to witness the result of a plan of treatment they might think proper to adopt.

In 1820, a law was passed, making it imperative upon the attending senior and junior surgeons, to give clinical lectures upon the cases under their care during the winter, and their period of attendance was extended to 12 months; the two surgeons who are elected in August attending only the consultations and operations till next May, when they commence their daily attendance, and continue till the ensuing May, when they give place to their successors, continuing to act as consulting surgeons till 1st November, when they cease to have any thing farther to do with the hospital, and cannot be re-elected for the two following years.

This was a still farther improvement; it is a plan of attendance highly liberal, conducive to the interests of the Infirmary, which it extends among the profession—useful to the profession itself, as it excites emulation amongst its members—diffuses more widely surgical knowledge—gives to the student, who must fee the house for two years, an opportunity of witnessing a greater variety of surgical treatment; and prevents all the evils of the close or monopolizing system. And its good effects are apparent; for it may, without vanity, be affirmed, that in no city in the kingdom, is surgery more ardently studied, or more successfully practised, than in Glasgow.

For the first twenty years after the commencement of the Glasgow Royal Infirmary, a similar system prevailed in the election of the Physicians or *medical* attendants, who, after having served for two years, retired, and were not again re-elected for two years, if others could be found to fill the situation; but the medical attendants being confined to what are called *pure physicians* who act as consulting physicians, that is, Doctors in Medicine who never acted as general practitioners, or, who having done so, or attempted to do so and failed, had given up the surgical or obstetrical department of the profession; or what amounted to much the same thing, if this had left them, they became pure physicians, and were considered eligible to serve as physicians to the hospital, while the general prac-

---

* Glasgow Journal, No. XV.

titioner, whose every day business it is to attend to all cases of disease, and who, of course, enjoys a more extensive opportunity of acquiring practical knowledge and experience, was excluded.

It is understood, that the present enlightened managers are about to put an end to this close system, and it may therefore be expected that an attempt will be made to set up the cry, that 'the profession is in danger,' and 'that nothing but ruin and revolution may be expected to follow such a rash and violent innovation;' that it is a blow aimed at the existence of the *genuine* physicians, 'calculated to level all distinctions in rank,' and uproot established privileges, hallowed by time; that it is an 'unwarrantable interference with vested rights,' 'contrary to the constitution, and the practice established by experience;' that it is an audacious attack upon a system which has hitherto 'worked well;' that 'it is, in fact, not reform, but revolution,' and can be looked at in no other light, 'than rapacity and robbery on the part of the general practitioners,' and a change which, by letting in a host of unqualified persons to act as medical attendants in the hospital, must be followed by the utter ruin of the profession, and of the best interests of the Infirmary.

The Directors will no doubt answer all this, by asserting, that by the original charter of the Royal Infirmary, they are not restricted in their elections to *pure* physicians, but only to those they consider best qualified to fill the situation; that they believe that the education of the M. D.'s who act as general practitioners, is at least equal to that of those who act only as physicians, and that from their being daily in the habit of attending to every variety of medical practice, they must have at least equal, if not superior knowledge, and experience of diseases, to those who are only called to visit and consult in extreme cases, chiefly with a view to satisfy those who are still so much under the influence of prejudices, as to suppose that there is a charm attached to the term physician, which gives those who assume it a kind of second sight; but that such notions are now confined to an antiquated race, 'are contrary to the spirit of the age,' and must soon disappear before the 'light of liberalism' and the 'march of intellect;' and that they, the managers, are quite capable of forming regulations, by which those without experience will be prevented from getting into the situation of medical attendants of the hospital."

### Injuries of the Head.

Five severe cases of this description were admitted under Dr. Perry, from November 1830, till May 1831.

*Case* 1. B. C. labourer, was admitted on the 20th May. At 5, p.m. of the 19th he fell, whilst intoxicated, from the top of a stair ten feet high. When raised he was quite insensible, and blood issued from the right ear. On admission he was comatose, and when loudly spoken to merely uttered some indistinct murmurs—eyes turgid—pupils slightly dilated—breathing natural—pulse 92, of moderate strength—no external injury of skull detected. He had been bled previous to admission. Cold lotions, leeches, purgatives, &c. were employed, but he lay with little change till the 22d, when he died.

*Dissection.* Over both hemispheres of the brain, beneath the dura mater, and at the base of the skull there existed a large quantity of coagulated blood. The brain was more vascular than natural. The anterior and left middle lobes were extensively and deeply lacerated, the torn parts filled with coagulated blood, and the medullary substance around this of the consistence of pulp. Several ounces of serum beneath the cerebellum, and a little in the lateral ventricles. The squamous portion of the temporal bone and greater wing of the sphenoid, were fractured horizontally, while another fracture took a direction along the petrous portion and sella turcica.

*Case* 2. J. S. æt. 35, admitted June 6th, in a state of complete insensibility, with stertorous breathing; pupils, particularly the right, dilated; bleeding from right ear and nostrils; extremities cold, pulse 66, weak and irregular; over superior posterior angle of parietal bone, a firm swelling of the size of half-a-crown, with small puffy tumours at outer canthus of both orbits; no fracture felt. He had been knocked down in the morning by a blow from a fist, and fell on the right side of the body on the pavement. When raised he was insensible, and so he continued; he had been bled. He died 14 hours after the receipt of the injury.

*Dissection.* "Skull found fractured below tumour on right side of head. Fracture extended from about the centre of the impression made by temporal muscle on parietal bone, to the middle of the squamous portion of the temporal bone, where it divided, one division going downwards and backwards to the middle part of the mastoid process, when it then ran forwards to the meatus auditorious externus; the other division of the fracture ran forwards and inwards, to foramen lacerum anterius. A portion of the parietal bone was comminuted and slightly depressed. The walls of the cranium, particularly the right parietal bone, were unusually thin, not half the thickness of an ordinary skull, and, when examined by the light, unusually diaphanous. Betwixt the parietal bone and dura mater, was a layer of coagulated blood, occupying a space of four inches square; the middle artery of the dura mater was ruptured, and a layer of blood effused betwixt the dura mater and tunica arachnoidea, covering the whole of the right hemisphere and a portion of left; about half an ounce at base of brain. The substance of the brain was healthy."

*Case* 3. J. Corbett, æt. 25, admitted at 7, a. m. of the 9th June, having been found at 4, a. m. lying in a state of stupor below a working steam-engine. The scalp on the right side of the head was separated from the muscles, so as to form a semicircular flap. The skull was fractured, but not depressed, about the posterior inferior angle of the parietal bone, the fracture appearing to extend over the crown of the head and downwards under the right temporal muscle. Immediately over the left temple was a puffy swelling, and under it apparent depression of bone. The left eyelids were much ecchymosed, the left clavicle fractured, and on the left side of the chest was a severe lacerated wound. He lay in a stupid state, was easily roused, but could give no distinct answer to questions. The right pupil dilated but contractile, the left not visible from ecchymosis; no vomiting, breathing natural, pulse 96 and soft. At 9, a. m. it was determined in consultation to trace the fracture further; it was found to extend towards the base of the skull; on puncturing the tumour on the left side the bone was thought to be depressed. Nothing further was attempted.

On the 10th he appeared more sensible, but rather restless, without heat of skin. *Hirud.* xij. *capiti—calomel,* gr. x. He was purged, and on the 11th was still more sensible, without fever. On the 14th the puffy tumour on the left temple was gone, and there was no appearance of depression, and the scalp-wound was suppurating. On the 18th a piece of bone, the size of a shilling, was denuded. The patient was now nearly sensible, but irritable, and voluntarily left the hospital. Two months afterwards he returned in good health, the portion of bone having exfoliated and left a space as large as a shilling, when the pulsations of the brain were seen. The treatment during his stay in the hospital had chiefly consisted in the exhibition of purgatives, and the application of cold lotions to the head.

"It is only necessary to place these cases in contrast, to make them interesting, as showing the difference betwixt compression of the brain and concussion. The first is a well marked case of concussion, joined with a certain degree of compression. The second a case of pure compression, and the third a case of pure concussion, though from the manner the blow appears to have been received, and the extensive fracture, compression might have been expected to exist. The last case shows, also, how little medical or surgical interference is necessary, when the constitution is otherwise good—here nothing was done except keeping the bowels open, and watching carefully the first appearance of inflammatory symptoms to act vigorously. This was fortunately not necessary.

It may be further remarked that, in the first case, the absence of the stertorous breathing, the regularity and moderate state of the pulse, the ability, to a certain extent, to use the voluntary muscles, without the power of directing them to any object, and the excitability of the involuntary muscles, might have led to the belief that the case was one of concussion; but the blood issuing from the ear, the loss of speech and of mind, indicated, along with this, either extravasation or serious injury of the brain, which was illustrated by the post mortem examination. While, in the second case, the complete insensibility to external impressions, the stertorous breathing, dilated pupils, irregular pulse, and coldness of the extremities, with the blood issuing from the ear, all immediately following the injury, indicated complete compression of the brain, and consequently the nearly total suspension of the action, of both voluntary and involuntary muscles. In the 3d case, though the patient lay for some days in a stupid state, he could easily be roused. Though incoherent, the action of the voluntary and involuntary muscles was perfect, as shewn by his exertions, and his getting out of bed when he felt the inclination to void urine. It will be remarked that, in the two first cases, the patients had been bled previous to admission, immediately after the receipt of the injury. This practice cannot be too severely reprobated, as there is no doubt, that in many cases, it has the effect of sinking the patient irrecoverably. It is true, that in the cases detailed, nothing could have saved the patients, but it might have been otherwise. In the third case, this practice might have been followed by serious consequences."

We hope that Dr. Perry will continue to favour the professional public with what cases of interest may fall under his observation.

---

